# Complete genome of *Achromobacter xylosoxidans* strain MUI-24198746 isolated from a 22-month-old child with persistent otorrhea

**DOI:** 10.1128/mra.01328-25

**Published:** 2026-08-26

**Authors:** Michael Berktold, Beatriz Daza Prieto, Miriam Govrins, Silke Huber, Philipp Grubwieser, Verena Fleischer, Mar Cubertorer Navarro, Tobias Mösenbacher, Elisabeth Edler, Franziska Jauernegger, Theresa Mayrhofer, Sandra Köberl-Jelovcan, Cornelia Lass-Flörl, Werner Ruppitsch, Joachim Schmutzhard

**Affiliations:** 1 Institute of Hygiene and Medical Microbiology, Medical University of Innsbruck 27280, Innsbruck, Austria; 2 Institute for Medical Microbiology and Hygiene, Austrian Agency for Health and Food Safety 31190, Vienna, Austria; 3 Department of Infectious Disease Diagnostics and Epidemiology, Center for Pathophysiology, Infectious Diseases, and Immunology, Medical University of Vienna 27258, Vienna, Austria; 4 Institute for Medical Microbiology and Hygiene, Austrian Agency for Health and Food Safety, Graz, Austria; 5 Faculty of Food Technology, Food Safety and Ecology, University of Donja Gorica 217861, Podgorica, Montenegro; 6 Department for Otorhinolaryngology, Head and Neck Surgery, Medical University of Innsbruck 27280, Innsbruck, Austria; University of Pittsburgh School of Medicine, Pittsburgh, Pennsylvania, USA

**Keywords:** *Achromobacter xylosoxidans*, otitis media, otorrhea, complete genome, infection, multi-resistance

## Abstract

*Achromobacter xylosoxidans* is an emerging pathogen exhibiting high levels of intrinsic antibiotic resistance. Here, we report the complete genome of *A. xylosoxidans* strain MUI-24198746 isolated from an ear swab originating from the right ear of a 22-month-old patient presenting with bilateral tympanic membrane perforation and persistent otorrhea.

## ANNOUNCEMENT

*Achromobacter xylosoxidans (Ax*) was first isolated from Japanese patients with chronic otitis media in 1971 (1). *Ax* is widespread in the environment, is resistant to many antimicrobials (2), and has emerged as an opportunistic pathogen causing various infections (3).

*Ax* MUI-24198746 was obtained from the ear of a 22-month-old girl with otitis media with effusion. Over 20 months, the girl developed bilateral tympanic membrane perforation and persistent otorrhea with *Ax*-positive smears. The ear swab was streaked on Columbia-III-blood-agar (Becton Dickinson, Austria) and incubated at 37°C for 48 h. *Ax* MUI-24198746 was identified by matrix-assisted laser desorption/ionization time-of-flight mass spectrometry (Biotyper library v4.1; Bruker Daltonics, Germany) and characterized by whole-genome sequencing.

Antimicrobial susceptibility testing was performed using disk diffusion according to the European Committee on Antimicrobial Susceptibility Testing guidelines (4). The minimal inhibitory concentrations were determined using gradient tests (Biomérieux, France; Liofilchem, Italy) and MIC Strips (Bruker, Germany) for colistin.

Genomic DNA was isolated from an overnight culture using MagNAPure24-Total-NA (Roche, Switzerland) and NucleoSpin Microbial-DNA (Macherey-Nagel, Germany) kits. The DNA Prep(M) kit was used for library preparation followed by 2 × 300 bp sequencing on a MiSeq (Illumina, USA). The Native Barcoding Kit 24 V14 (SQK-NBD114.24, Oxford Nanopore Technologies [ONT]) with the long fragment buffer for fragment enrichment above 3 kb, and a FLO-MIN114 R10.4.1 SpotON flow cell were used for long-read sequencing on a GridION (ONT, UK) (5).

A total of 578,172 ONT reads were obtained using Dorado v7.1.4 in Superior base-calling mode and filtered using Filtlong v0.2.1 (min_length, 1,000; keep percent, 90; and target_bases, 500,000,000). A total of 3,668,972 Illumina reads were visually quality controlled using FastQC v0.11.9 (6) and used untrimmed for hybrid assembly with Hybracter v0.11.1 (7) via its Docker container hosted at Quay.io (https://quay.io/repository/gbouras13/hybracter; image tag: 0.11.1), which employs Flye v2.9.5-b1801 for long-read assembly (8). Hybracter was run with the --auto flag, which estimates the genome size from long-read data using lrge (9) and assesses completeness on this estimate. The final assembly consisted of a single complete and circular contig with a mean coverage of 83-fold and a total length of 6,757,409 bp. (Table 1). Circularity was confirmed by Flye, and the chromosome was subsequently reoriented using Dnaapler (10) to start at *dnaA*.

**TABLE 1:** Characteristics of *A. xylosoxidans* MUI-24198746

Attribute	Finding
Assembly size (bp)	6,757,409
No. of contigs	1
Hybrid assembly genome coverage (x)	83
No. of short reads	3,668,972
No. of long reads	578,172
Long-read fragment N50 (bp)	8,128
GC content (%)	67.52
Total no. of genes	6,243
Pseudogenes	41
Total no. of coding sequences	6,172
No. of RNA genes	71
No. of tRNAs	57
No. of complete rRNAs (5S, 16S, 23S)	4, 3, 3
No. of ncRNAs	4
Total no. of pseudogenes	41
No. of CRISPR/Cas elements	5
No. of plasmids/ mobile genetic elements	0/IS*Rme15*
No. of phages/ prophages	two intact: PHAGE_Burkho_KS14 (31.5 Kb, GC 64.06%) NC_015273, PHAGE_Burkho_KS9 (30.2 Kb, GC 65.87%) NC_013055 two questionable: PHAGE_Bordet_BPP_1 (42.9 Kb, GC 61.46%) NC_005357, PHAGE_Salmon_SEN34 (16.5 Kb, GC 66.73%) NC_028699
Sequence type (ST)	5
Antimicrobial resistance gene (ARG, perfect and strict hits)^*a*^	*bla*_OXA-114h_ (100%), *bla*_AXC-1_ (99.66%), *catB3* (80.98%), antibiotic efflux: *adeF* (81.59%), *qacG* (47.17%), *oprZ* (96.33%), and *axyY* (98.56%)
Minimum inhibitory concentration (MIC)	>256 mg/L for aztreonam, cefepime, and ceftolozane/tazobactam; 4 mg/L for ceftazidime; 2 mg/L for ceftazidime/avibactam; 1 mg/L for colistin

^*a*^ In brackets: % identity to reference in CARD database.

The PubMLST (11) *Achromobacter* sp. multilocus-sequence-typing scheme (12), CARD v4.0.1 (13), PlasmidFinder v2.0.1 (14), MobileElementFinder v1.0.3 (15), and PHASTEST (16) were used to determine the sequence type (ST) to detect AMR targets, plasmids, mobile genetic elements, and phages. Default software parameters were used unless otherwise specified.

Genome annotation using NCBI’s Prokaryotic Genome Annotation Pipeline v6.11 (17) identified 6,243 genes, 6,172 coding sequences, and 71 RNA genes (Table 1). The Basic Local Alignment Search Tool v2.15.0 (18) and digital DNA-DNA hybridization using the Type (Strain) Genome Server (19) revealed 100% 16S rRNA and 88.9% genome identity with type strain NRBC15126^T^.

MUI-24198746 belongs to ST5, carries five CRISPR elements, two IS*Rme15* insertion sequences (IS), the beta-lactamases OXA-114h and AXC-1, and three efflux pumps (Table 1). The strain was phenotypically resistant to all antimicrobial agents using EUCAST breakpoints (where available), except cefiderocol. MIC values are shown in Table 1.

## Data Availability

This Whole Genome Shotgun project has been deposited in NCBI GenBank under the accession number PRJNA1336156. The version described in this paper is the first version, JBRILF000000000. Illumina raw sequence reads have been deposited in the Sequence Read Archive (SRA) under the accession number SRR35659261 and Oxford Nanopore Technologies reads under SRA accession number SRR35674568.
